# (Dimethyl sulfoxide-κ*O*)di­phenyl­(3-thioxo-3*H*-1,2-dithiole-4,5-dithiol­ato-κ^2^ *S* ^4^,*S* ^5^)tin(IV)

**DOI:** 10.1107/S1600536809047904

**Published:** 2009-11-18

**Authors:** Nadia M. Comerlato, Edward R. T. Tiekink, James L. Wardell, Solange M. S. V. Wardell

**Affiliations:** a Departamento de Química Inorgânica, Instituto de Química, Universidade, Federal do Rio de Janeiro, CP 68563, 21941-909 Rio de Janeiro, RJ, Brazil; b Department of Chemistry, University of Malaya, 50603 Kuala Lumpur, Malaysia; c Centro de Desenvolvimento Tecnológico em Saúde (CDTS), Fundação Oswaldo Cruz (FIOCRUZ), Casa Amarela, Campus de Manguinhos, Av. Brasil 4365, 21040-900, Rio de Janeiro, RJ, Brazil; d CHEMSOL, 1 Harcourt Road, Aberdeen AB15 5NY, Scotland

## Abstract

The Sn atom in the title compound, [Sn(C_6_H_5_)_2_(C_3_S_5_)(C_2_H_6_OS)], exists within a distorted trigonal-bipyramidal geometry defined by two S atoms of the 1,2-dithiole-3-thione-4,5-dithiol­ate dianion, two *ipso*-C atoms from the phenyl groups, and the O atom of the dimethyl sulfoxide mol­ecule. In this description, one of the S atoms and the O occupy axial positions. In the crystal, centrosymmetrically related mol­ecules associate *via* pairs of C—H⋯S contacts, forming dimeric aggregates.

## Related literature

For background to the synthesis of dmt compounds, see: Steimecke *et al.* (1982 ▶). For related crystal structures, see: Aupers *et al.* (1998 ▶); Khan *et al.* (1998 ▶); Chohan *et al.* (1999 ▶); Bordinhão *et al.* (2006 ▶, 2008 ▶); Comerlato *et al.* (2008 ▶). For additional analysis of geometry, see: Addison *et al.* (1984 ▶).
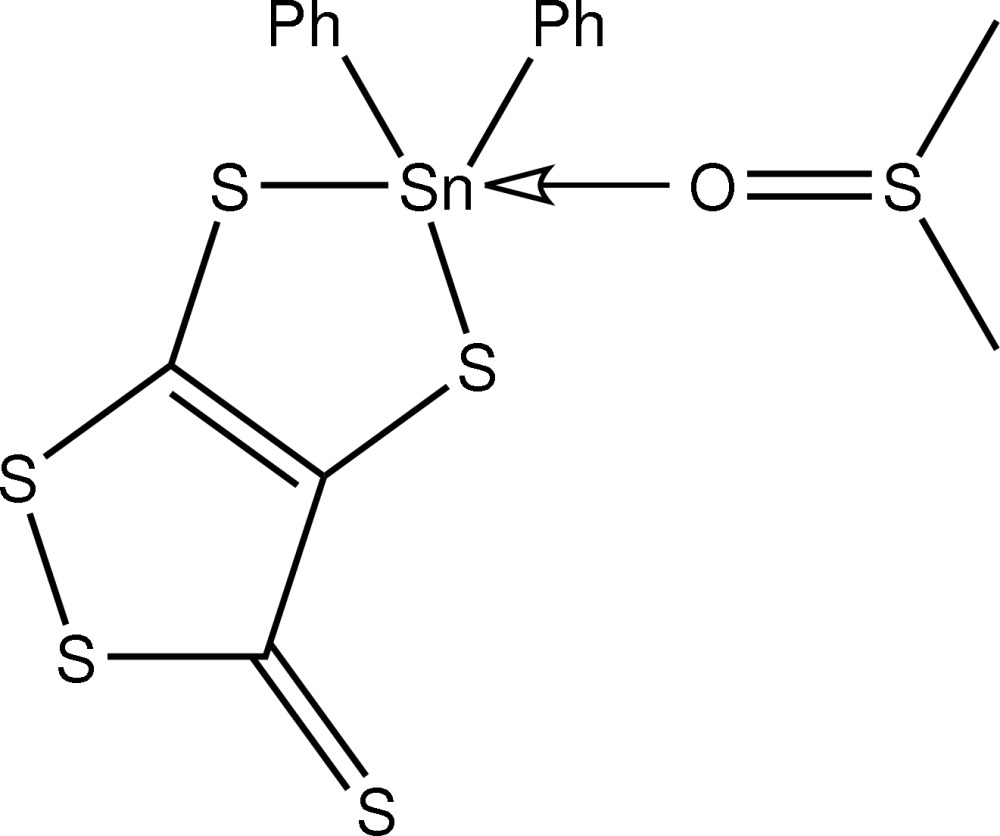


## Experimental

### 

#### Crystal data

[Sn(C_6_H_5_)_2_(C_3_S_5_)(C_2_H_6_OS)]
*M* *_r_* = 547.35
Monoclinic, 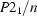

*a* = 11.1420 (5) Å
*b* = 15.7237 (6) Å
*c* = 11.9646 (6) Å
β = 96.892 (2)°
*V* = 2080.97 (16) Å^3^
*Z* = 4
Mo *K*α radiation
μ = 1.83 mm^−1^
*T* = 120 K
0.24 × 0.16 × 0.10 mm

#### Data collection

Bruker–Nonius 95mm CCD camera on κ-goniostat diffractometer
Absorption correction: multi-scan (*SADABS*; Sheldrick, 2007 ▶) *T*_min_ = 0.536, *T*_max_ = 0.746
22729 measured reflections
4770 independent reflections
3906 reflections with *I* > 2σ(*I*)
*R*_int_ = 0.057

#### Refinement

*R*[*F* ^2^ > 2σ(*F* ^2^)] = 0.037
*wR*(*F* ^2^) = 0.088
*S* = 1.06
4770 reflections
228 parameters
H-atom parameters constrained
Δρ_max_ = 0.85 e Å^−3^
Δρ_min_ = −1.26 e Å^−3^

### 

Data collection: *COLLECT* (Hooft, 1998 ▶); cell refinement: *DENZO* (Otwinowski & Minor, 1997 ▶) and *COLLECT*; data reduction: *DENZO* and *COLLECT*; program(s) used to solve structure: *SHELXS97* (Sheldrick, 2008 ▶); program(s) used to refine structure: *SHELXL97* (Sheldrick, 2008 ▶); molecular graphics: *DIAMOND* (Brandenburg, 2006 ▶); software used to prepare material for publication: *publCIF* (Westrip, 2009 ▶).

## Supplementary Material

Crystal structure: contains datablocks global, I. DOI: 10.1107/S1600536809047904/hb5220sup1.cif

Structure factors: contains datablocks I. DOI: 10.1107/S1600536809047904/hb5220Isup2.hkl

Additional supplementary materials:  crystallographic information; 3D view; checkCIF report

## Figures and Tables

**Table 1:** Selected bond lengths (Å)

Sn—C4	2.130 (3)
Sn—C10	2.133 (3)
Sn—O1	2.311 (2)
Sn—S1	2.4357 (9)
Sn—S2	2.5582 (9)

**Table 2:** Hydrogen-bond geometry (Å, °)

*D*—H⋯*A*	*D*—H	H⋯*A*	*D*⋯*A*	*D*—H⋯*A*
C6—H6⋯S2^i^	0.95	2.71	3.599 (4)	157

Symmetry code: (i) 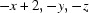.

**Fig. 1.**
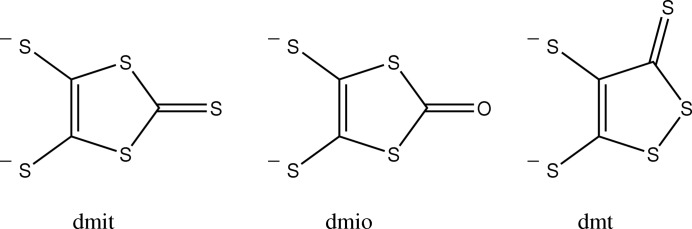
Preparation of the title compound.

**Fig. 2.**
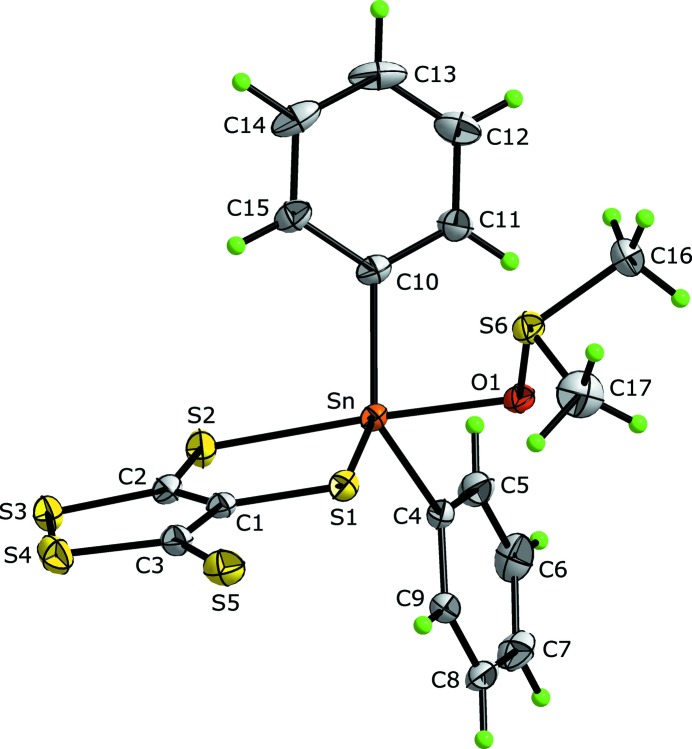
Molecular structure (I) showing displacement ellipsoids at the 50% probability level.

**Fig. 3.**
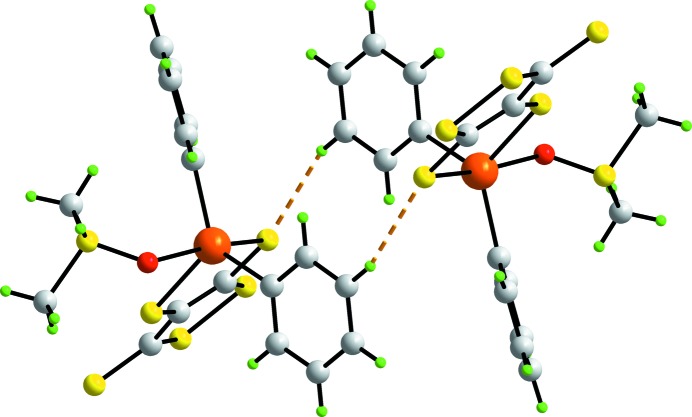
Supramolecular dimer in (I) mediated by C–H···S contacts (orange dashed lines). Colour code: Sn, orange; S, yellow;O, red; C, grey; and H, green.

## supplementary crystallographic information

### Comment

While several structures of (1,3-dithiole-2-thione-4,5-dithiolato)tin, [Sn-dmit], and (1,3-dithiole-2-one-4,5-dithiolato)tin [Sn-dmio] complexes have been reported (*e.g.*, Comerlato *et al.*, 2008), similar (1,2-dithiole-3-thione-4,5-dithiolato)tin complexes have been essentially neglected, with only one systematic study known (Bordinhão *et al.*, 2006; Bordinhão *et al.*, 2008); see Fig. 1 for chemical structures of dmit, dmio and dmt. The poor solubility of 1,2-dithiole-3-thione-4,5-dithiolato (dmt) complexes has been put forward as a major cause for the limited number of their reported crystal structures. Attempts to obtain good crystals of Ph_2_Sn(dmt), prepared from reaction of Na_2_(dmt) and Ph_2_SnCl_2_ in MeOH solution, failed as only amorphous material was obtained. However, crystallization of Ph_2_Sn(dmt) from a DMSO/MeOH solution produced crystals of the DMSO solvate, (I), suitable for the X-Ray study reported herein.

As compounds, *R*_2_Sn(dmit) and *R*_2_Sn(dmio), having non-functionalized alkyl or aryl *R* groups (*e.g.*, *R* = Me, Et, Bu or Ph), are aggregated in both the solid–state and in non-coordinating solvents as a consequence of intermolecular Sn···S interactions., it is assumed that the *R*_2_Sn(dmt) analogues are similarly aggregated. The formation of adducts such as [Ph_2_Sn(dmt)(dmso)] will generally provide coordinatively saturated tin centres and hence result in appreciably more soluble species having essentially non-interacting cations and anions. Structures of ionic species, [Q][*R*_2_Sn(dmit)*X*] and [Q][*R*_2_Sn(dmio)*X*] [Q+ = onium cation, *X* = halide or pseudohalide], with 5-coordinate tin have also been determined (Chohan *et al.*, 1999; Khan *et al.*, 1998; Aupers *et al.*, 1998).

The Sn atom in (1) is five-coordinate, existing within a C_2_OS_2_ donor set defined by a chelating dmt ligand, two *ipso*-C atoms and the O atom derived from the DMSO molecule, Fig. 2. The coordination geometry is based on a trigonal bipyramid with the S2–Sn–O1 axial angle being 166.52 (6) °. As expected, the Sn–S1_equatorial_ distance of 2.4357 (9) Å is shorter than the Sn–S2_axial_ distance of 2.5582 (9) Å. The coordination geometry is distorted towards trigonal bipyramidal (TP). This is quantified by the value of τ = 0.72, which compares with the ideal values of 1.0 and 0.0 for TP and square pyramidal, respectively (Addison *et al.*, 1984).

The most prominent intermolecular interaction connecting molecules is of the type C–H···S and these occur between centrosymmetric pairs to form loosely associated dimers, Table 1 and Fig. 3.

### Experimental

To a stirred suspension of 4,5-bis(benzoylthio)-1,2-dithiole-3-thione (Steimecke *et al.*, 1982) (410 mg, 1 mmol) in methanol (10 ml), under argon, was added a sodium methoxide solution prepared from sodium (150 mg, 6.75 mmol) and methanol (10 ml). To the resulting purple solution of Na_2_dmt was added with stirring a methanolic solution of Ph_2_SnCl_2_ (345 mg, 1 mmol). The reaction mixture was stirred for 1 h, rotary evaporated and the residue washed well with water. The solid residue (535 mg) was dissolved in a mixture of DMSO and MeOH (*ca* v:v 3:1) and left to slowly recrystallize to give (I); m.pt. 428–431 K (dec.) IR (KBr, cm^-1^): 1061 (ν C—S), 950, 941 (ν S—O), 910, 827, 720 (ν C=S).

### Refinement

All H atoms were geometrically placed (C–H = 0.95–0.98 Å) and refined as riding with *U*_iso_(H) = 1.2–1.5*U*_eq_(C). The maximum and minimum residual electron density peaks of 0.85 and 1.26 e Å^-3^, respectively, were located 1.81 Å and 0.82 Å from the S1 and Sn atoms, respectively.

### Crystal data

**Table:**

[Sn(C_6_H_5_)_2_(C_3_S_5_)(C_2_H_6_OS)]	*F*(000) = 1088
*M**_r_* = 547.35	*D*_x_ = 1.747 Mg m^−^^3^
Monoclinic, *P*2_1_/*n*	Mo *K*α radiation, λ = 0.71069 Å
Hall symbol: -P 2yn	Cell parameters from 44319 reflections
*a* = 11.1420 (5) Å	θ = 2.9–27.5°
*b* = 15.7237 (6) Å	µ = 1.83 mm^−^^1^
*c* = 11.9646 (6) Å	*T* = 120 K
β = 96.892 (2)°	Block, yellow
*V* = 2080.97 (16) Å^3^	0.24 × 0.16 × 0.10 mm
*Z* = 4	

### Data collection

**Table:**

Bruker–Nonius 95mm CCD camera on κ-goniostat diffractometer	4770 independent reflections
Radiation source: Bruker-Nonius FR591 rotating anode	3906 reflections with *I* > 2σ(*I*)
graphite	*R*_int_ = 0.057
Detector resolution: 9.091 pixels mm^-1^	θ_max_ = 27.5°, θ_min_ = 3.1°
φ and ω scans	*h* = −14→14
Absorption correction: multi-scan (*SADABS*; Sheldrick, 2007)	*k* = −20→19
*T*_min_ = 0.536, *T*_max_ = 0.746	*l* = −15→15
22729 measured reflections	

### Refinement

**Table:**

Refinement on *F*^2^	Primary atom site location: structure-invariant direct methods
Least-squares matrix: full	Secondary atom site location: difference Fourier map
*R*[*F*^2^ > 2σ(*F*^2^)] = 0.037	Hydrogen site location: inferred from neighbouring sites
*wR*(*F*^2^) = 0.088	H-atom parameters constrained
*S* = 1.06	*w* = 1/[σ^2^(*F*_o_^2^) + (0.0404*P*)^2^ + 1.7706*P*] where *P* = (*F*_o_^2^ + 2*F*_c_^2^)/3
4770 reflections	(Δ/σ)_max_ < 0.001
228 parameters	Δρ_max_ = 0.85 e Å^−^^3^
0 restraints	Δρ_min_ = −1.26 e Å^−^^3^

### Special details

**Table:**

Geometry. All s.u.'s (except the s.u. in the dihedral angle between two l.s. planes) are estimated using the full covariance matrix. The cell s.u.'s are taken into account individually in the estimation of s.u.'s in distances, angles and torsion angles; correlations between s.u.'s in cell parameters are only used when they are defined by crystal symmetry. An approximate (isotropic) treatment of cell s.u.'s is used for estimating s.u.'s involving l.s. planes.
Refinement. Refinement of *F*^2^ against ALL reflections. The weighted *R*-factor *wR* and goodness of fit *S* are based on *F*^2^, conventional *R*-factors *R* are based on *F*, with *F* set to zero for negative *F*^2^. The threshold expression of *F*^2^ > 2σ(*F*^2^) is used only for calculating *R*-factors(gt) *etc*. and is not relevant to the choice of reflections for refinement. *R*-factors based on *F*^2^ are statistically about twice as large as those based on *F*, and *R*- factors based on ALL data will be even larger.

### Fractional atomic coordinates and isotropic or equivalent isotropic displacement parameters (Å^2^)

**Table:**

	*x*	*y*	*z*	*U*_iso_*/*U*_eq_	
Sn	0.823308 (19)	0.079364 (13)	0.201486 (19)	0.01886 (9)	
S1	0.64254 (8)	0.07006 (5)	0.29688 (7)	0.02196 (18)	
S2	0.68225 (8)	0.10256 (6)	0.01905 (8)	0.0263 (2)	
S3	0.42347 (8)	0.12813 (5)	−0.02069 (8)	0.0266 (2)	
S4	0.30214 (8)	0.12492 (6)	0.09713 (8)	0.0302 (2)	
S5	0.35612 (8)	0.08574 (6)	0.33685 (9)	0.0301 (2)	
S6	0.89763 (8)	0.13614 (5)	0.46902 (8)	0.0264 (2)	
O1	0.9096 (2)	0.06287 (13)	0.38570 (19)	0.0218 (5)	
C1	0.5279 (3)	0.09377 (19)	0.1865 (3)	0.0210 (7)	
C2	0.5481 (3)	0.1063 (2)	0.0763 (3)	0.0226 (7)	
C3	0.4068 (3)	0.0995 (2)	0.2126 (3)	0.0249 (7)	
C4	0.8953 (3)	−0.0427 (2)	0.1706 (3)	0.0199 (7)	
C5	1.0061 (3)	−0.0547 (2)	0.1308 (3)	0.0291 (8)	
H5	1.0562	−0.0073	0.1200	0.035*	
C6	1.0440 (3)	−0.1366 (2)	0.1067 (3)	0.0349 (9)	
H6	1.1200	−0.1448	0.0798	0.042*	
C7	0.9712 (3)	−0.2061 (2)	0.1217 (3)	0.0327 (9)	
H7	0.9965	−0.2616	0.1037	0.039*	
C8	0.8622 (3)	−0.1945 (2)	0.1626 (3)	0.0277 (8)	
H8	0.8126	−0.2423	0.1735	0.033*	
C9	0.8241 (3)	−0.1133 (2)	0.1883 (3)	0.0216 (7)	
H9	0.7495	−0.1059	0.2179	0.026*	
C10	0.9274 (3)	0.1927 (2)	0.1953 (3)	0.0212 (7)	
C11	1.0477 (3)	0.1947 (2)	0.2438 (3)	0.0260 (7)	
H11	1.0842	0.1443	0.2758	0.031*	
C12	1.1145 (3)	0.2691 (2)	0.2459 (3)	0.0331 (9)	
H12	1.1963	0.2698	0.2792	0.040*	
C13	1.0608 (4)	0.3427 (2)	0.1990 (4)	0.0376 (10)	
H13	1.1055	0.3943	0.2023	0.045*	
C14	0.9438 (4)	0.3415 (2)	0.1480 (3)	0.0350 (9)	
H14	0.9085	0.3919	0.1147	0.042*	
C15	0.8763 (3)	0.2666 (2)	0.1450 (3)	0.0270 (8)	
H15	0.7956	0.2658	0.1087	0.032*	
C16	1.0469 (3)	0.1532 (2)	0.5361 (3)	0.0351 (9)	
H16A	1.0823	0.0987	0.5628	0.053*	
H16B	1.0443	0.1917	0.6002	0.053*	
H16C	1.0962	0.1785	0.4824	0.053*	
C17	0.8353 (4)	0.0869 (3)	0.5831 (4)	0.0423 (10)	
H17A	0.7533	0.0670	0.5574	0.064*	
H17B	0.8326	0.1281	0.6441	0.064*	
H17C	0.8858	0.0384	0.6105	0.064*	

### Atomic displacement parameters (Å^2^)

**Table:**

	*U*^11^	*U*^22^	*U*^33^	*U*^12^	*U*^13^	*U*^23^
Sn	0.01908 (13)	0.01369 (12)	0.02314 (14)	0.00039 (8)	−0.00013 (9)	0.00187 (8)
S1	0.0222 (4)	0.0193 (4)	0.0241 (5)	0.0009 (3)	0.0015 (3)	0.0028 (3)
S2	0.0210 (4)	0.0330 (5)	0.0243 (5)	0.0008 (3)	0.0000 (4)	0.0059 (4)
S3	0.0213 (4)	0.0276 (4)	0.0293 (5)	0.0011 (3)	−0.0039 (4)	0.0000 (4)
S4	0.0194 (4)	0.0347 (5)	0.0356 (5)	0.0019 (4)	−0.0007 (4)	−0.0036 (4)
S5	0.0259 (5)	0.0298 (5)	0.0360 (6)	−0.0008 (3)	0.0096 (4)	−0.0007 (4)
S6	0.0326 (5)	0.0183 (4)	0.0263 (5)	0.0038 (3)	−0.0041 (4)	−0.0022 (3)
O1	0.0270 (13)	0.0153 (11)	0.0224 (13)	0.0015 (9)	0.0004 (10)	−0.0005 (9)
C1	0.0199 (16)	0.0128 (15)	0.0292 (19)	0.0002 (12)	−0.0021 (14)	−0.0015 (13)
C2	0.0222 (17)	0.0168 (15)	0.0276 (19)	0.0021 (13)	−0.0011 (14)	−0.0005 (13)
C3	0.0210 (17)	0.0190 (16)	0.034 (2)	−0.0001 (13)	−0.0012 (15)	−0.0006 (14)
C4	0.0210 (16)	0.0172 (15)	0.0203 (17)	0.0019 (13)	−0.0024 (13)	0.0010 (13)
C5	0.0271 (19)	0.0246 (17)	0.036 (2)	0.0000 (14)	0.0059 (16)	0.0053 (16)
C6	0.031 (2)	0.041 (2)	0.035 (2)	0.0111 (17)	0.0121 (17)	0.0045 (17)
C7	0.044 (2)	0.0218 (17)	0.032 (2)	0.0098 (16)	0.0041 (17)	−0.0024 (15)
C8	0.039 (2)	0.0174 (16)	0.0254 (19)	−0.0013 (14)	0.0002 (16)	0.0044 (14)
C9	0.0221 (17)	0.0195 (16)	0.0222 (18)	−0.0016 (13)	−0.0013 (14)	0.0008 (13)
C10	0.0257 (17)	0.0170 (15)	0.0216 (18)	−0.0003 (13)	0.0063 (14)	−0.0002 (13)
C11	0.0243 (17)	0.0233 (17)	0.030 (2)	−0.0018 (14)	0.0027 (15)	−0.0018 (14)
C12	0.029 (2)	0.034 (2)	0.038 (2)	−0.0086 (16)	0.0099 (17)	−0.0065 (17)
C13	0.049 (3)	0.0236 (18)	0.045 (2)	−0.0145 (17)	0.026 (2)	−0.0081 (17)
C14	0.050 (2)	0.0178 (17)	0.041 (2)	0.0041 (16)	0.021 (2)	0.0026 (16)
C15	0.0300 (19)	0.0197 (16)	0.032 (2)	0.0039 (14)	0.0051 (15)	0.0014 (14)
C16	0.035 (2)	0.033 (2)	0.034 (2)	−0.0047 (16)	−0.0112 (17)	0.0022 (17)
C17	0.049 (3)	0.049 (3)	0.031 (2)	−0.0049 (19)	0.012 (2)	−0.0018 (18)

### Geometric parameters (Å, °)

**Table:**

Sn—C4	2.130 (3)	C7—C8	1.375 (5)
Sn—C10	2.133 (3)	C7—H7	0.9500
Sn—O1	2.311 (2)	C8—C9	1.392 (5)
Sn—S1	2.4357 (9)	C8—H8	0.9500
Sn—S2	2.5582 (9)	C9—H9	0.9500
S1—C1	1.764 (3)	C10—C11	1.396 (5)
S2—C2	1.718 (3)	C10—C15	1.398 (5)
S3—C2	1.734 (3)	C11—C12	1.386 (5)
S3—S4	2.0674 (13)	C11—H11	0.9500
S4—C3	1.744 (4)	C12—C13	1.390 (6)
S5—C3	1.667 (4)	C12—H12	0.9500
S6—O1	1.540 (2)	C13—C14	1.372 (6)
S6—C16	1.778 (4)	C13—H13	0.9500
S6—C17	1.781 (4)	C14—C15	1.395 (5)
C1—C2	1.379 (5)	C14—H14	0.9500
C1—C3	1.423 (5)	C15—H15	0.9500
C4—C5	1.388 (5)	C16—H16A	0.9800
C4—C9	1.395 (5)	C16—H16B	0.9800
C5—C6	1.396 (5)	C16—H16C	0.9800
C5—H5	0.9500	C17—H17A	0.9800
C6—C7	1.384 (5)	C17—H17B	0.9800
C6—H6	0.9500	C17—H17C	0.9800
			
C4—Sn—C10	121.97 (12)	C6—C7—H7	120.0
C4—Sn—O1	86.68 (10)	C7—C8—C9	120.3 (3)
C10—Sn—O1	87.79 (10)	C7—C8—H8	119.8
C4—Sn—S1	112.16 (9)	C9—C8—H8	119.8
C10—Sn—S1	123.38 (9)	C8—C9—C4	120.2 (3)
O1—Sn—S1	79.58 (6)	C8—C9—H9	119.9
C4—Sn—S2	100.73 (9)	C4—C9—H9	119.9
C10—Sn—S2	97.58 (9)	C11—C10—C15	118.8 (3)
O1—Sn—S2	166.52 (6)	C11—C10—Sn	120.2 (2)
S1—Sn—S2	87.16 (3)	C15—C10—Sn	121.0 (2)
C1—S1—Sn	101.66 (12)	C12—C11—C10	120.9 (3)
C2—S2—Sn	98.03 (12)	C12—C11—H11	119.6
C2—S3—S4	94.35 (12)	C10—C11—H11	119.6
C3—S4—S3	96.65 (12)	C11—C12—C13	119.5 (4)
O1—S6—C16	104.84 (17)	C11—C12—H12	120.3
O1—S6—C17	104.05 (17)	C13—C12—H12	120.3
C16—S6—C17	98.6 (2)	C14—C13—C12	120.5 (3)
S6—O1—Sn	118.36 (12)	C14—C13—H13	119.7
C2—C1—C3	117.9 (3)	C12—C13—H13	119.7
C2—C1—S1	124.0 (3)	C13—C14—C15	120.3 (3)
C3—C1—S1	118.0 (3)	C13—C14—H14	119.9
C1—C2—S2	128.8 (3)	C15—C14—H14	119.9
C1—C2—S3	117.3 (3)	C14—C15—C10	120.0 (3)
S2—C2—S3	113.9 (2)	C14—C15—H15	120.0
C1—C3—S5	128.2 (3)	C10—C15—H15	120.0
C1—C3—S4	113.7 (3)	S6—C16—H16A	109.5
S5—C3—S4	118.1 (2)	S6—C16—H16B	109.5
C5—C4—C9	119.3 (3)	H16A—C16—H16B	109.5
C5—C4—Sn	123.5 (2)	S6—C16—H16C	109.5
C9—C4—Sn	117.2 (2)	H16A—C16—H16C	109.5
C4—C5—C6	120.0 (3)	H16B—C16—H16C	109.5
C4—C5—H5	120.0	S6—C17—H17A	109.5
C6—C5—H5	120.0	S6—C17—H17B	109.5
C7—C6—C5	120.3 (3)	H17A—C17—H17B	109.5
C7—C6—H6	119.9	S6—C17—H17C	109.5
C5—C6—H6	119.9	H17A—C17—H17C	109.5
C8—C7—C6	119.9 (3)	H17B—C17—H17C	109.5
C8—C7—H7	120.0		
			
C4—Sn—S1—C1	−105.26 (14)	O1—Sn—C4—C5	−94.0 (3)
C10—Sn—S1—C1	92.39 (15)	S1—Sn—C4—C5	−171.4 (3)
O1—Sn—S1—C1	172.71 (12)	S2—Sn—C4—C5	97.3 (3)
S2—Sn—S1—C1	−4.83 (10)	C10—Sn—C4—C9	173.5 (2)
C4—Sn—S2—C2	116.74 (14)	O1—Sn—C4—C9	88.2 (3)
C10—Sn—S2—C2	−118.59 (14)	S1—Sn—C4—C9	10.8 (3)
O1—Sn—S2—C2	−5.7 (3)	S2—Sn—C4—C9	−80.5 (2)
S1—Sn—S2—C2	4.72 (11)	C9—C4—C5—C6	1.4 (5)
C2—S3—S4—C3	−1.58 (16)	Sn—C4—C5—C6	−176.4 (3)
C16—S6—O1—Sn	−130.43 (17)	C4—C5—C6—C7	0.3 (6)
C17—S6—O1—Sn	126.56 (19)	C5—C6—C7—C8	−1.3 (6)
C4—Sn—O1—S6	177.97 (16)	C6—C7—C8—C9	0.5 (6)
C10—Sn—O1—S6	55.76 (16)	C7—C8—C9—C4	1.2 (5)
S1—Sn—O1—S6	−68.78 (13)	C5—C4—C9—C8	−2.2 (5)
S2—Sn—O1—S6	−58.2 (3)	Sn—C4—C9—C8	175.8 (3)
Sn—S1—C1—C2	4.4 (3)	C4—Sn—C10—C11	−42.9 (3)
Sn—S1—C1—C3	−176.2 (2)	O1—Sn—C10—C11	41.8 (3)
C3—C1—C2—S2	−179.4 (3)	S1—Sn—C10—C11	117.8 (2)
S1—C1—C2—S2	0.0 (5)	S2—Sn—C10—C11	−150.6 (3)
C3—C1—C2—S3	0.1 (4)	C4—Sn—C10—C15	138.4 (3)
S1—C1—C2—S3	179.45 (17)	O1—Sn—C10—C15	−136.9 (3)
Sn—S2—C2—C1	−4.1 (3)	S1—Sn—C10—C15	−60.9 (3)
Sn—S2—C2—S3	176.36 (15)	S2—Sn—C10—C15	30.7 (3)
S4—S3—C2—C1	1.1 (3)	C15—C10—C11—C12	2.3 (5)
S4—S3—C2—S2	−179.35 (16)	Sn—C10—C11—C12	−176.4 (3)
C2—C1—C3—S5	178.8 (3)	C10—C11—C12—C13	−0.1 (6)
S1—C1—C3—S5	−0.6 (4)	C11—C12—C13—C14	−1.8 (6)
C2—C1—C3—S4	−1.6 (4)	C12—C13—C14—C15	1.4 (6)
S1—C1—C3—S4	179.03 (17)	C13—C14—C15—C10	0.9 (5)
S3—S4—C3—C1	2.0 (2)	C11—C10—C15—C14	−2.7 (5)
S3—S4—C3—S5	−178.38 (18)	Sn—C10—C15—C14	176.0 (3)
C10—Sn—C4—C5	−8.7 (4)		

### Hydrogen-bond geometry (Å, °)

**Table:**

*D*—H···*A*	*D*—H	H···*A*	*D*···*A*	*D*—H···*A*
C6—H6···S2^i^	0.95	2.71	3.599 (4)	157

Symmetry codes: (i) −*x*+2, −*y*, −*z*.
